# Functional Outcome of Intertrochanteric Fractures of the Femur Treated With Standard Surgical Modalities

**DOI:** 10.7759/cureus.70909

**Published:** 2024-10-05

**Authors:** Harshad Argekar, Ganesh Dole, Mahesh Shinde, Mihir Patel, Yogeshwari H Patil, Atharva Sharma, Kshitij Sarwey, Renema Datta, Arnav Modi, Sakshi Sathe

**Affiliations:** 1 Orthopaedics, Hinduhridaysamrat Balasaheb Thackeray Medical College and Dr. R.N. Cooper Muncipal General Hospital, Mumbai, IND

**Keywords:** harris hip score, intertrochanteric femur fracture, intramedullary fixation, proximal femur nail, varus deformity

## Abstract

Introduction: Among the most prevalent injuries among the elderly with trivial trauma are trochanteric fractures. Osteoporosis and female sex are additional risk factors. The patient's age, the fracture, their overall health, the amount of time between the fracture and treatment, the effectiveness of the treatment, any concurrent medical care, and the stability of the fixation all affect how well the patient responds to treatment. For its fixation, various implants are used. The main aim of this study is to assess the functional outcome in intertrochanteric fractures using the Harris Hip Scoring in intertrochanteric fractures.

Method: Patients presenting with intertrochanteric femur fracture were evaluated. Written consent was taken from the patient. Radiographic data were collected. Complications and varus collapse evaluated. Functional outcome was assessed with the Harris Hip Scoring system.

Results: The proportion of excellent Harris Hip Evaluation (HHE) scores was the highest in proximal femur nail (PFNA2) at six months follow-up examination, which was 11 (57.19%) more than any method. In the incidence of varus deformity, the proportion was maximum in the case of the dynamic hip screw (DHS) with three patients (33.33%), followed by PFN in 11 patients (28.95%), and the least for PFNA2 in three patients (15.79%).

Conclusion: The mean operative time was lower in the PFN A2 group, and the HHE score values were higher than those of PFN and DHS. This study concludes that these implants have a comparable radiological and functional outcome for unstable intertrochanteric fracture except for less surgical time and blood loss in PFN A2.

## Introduction

The most common fractures in old age are intertrochanteric femur fractures. Women with osteoporosis are more likely to experience this kind of fracture, occurring three to four times more often than in men; typically, a minor fall is the cause [1]. This type of fracture can lead to death for certain people, as it can cause cardiac, pulmonary, or renal complications that are fatal. Within a year of experiencing an intertrochanteric fracture, about 10-30% of patients do not survive [2]. The frequency of intertrochanteric fractures has increased because of the increasing age of modern human populations [3]. In 1990, about 26% of hip fracture cases were seen in Asia alone. By 2025, this figure could rise to 37% and up to 45% by 2050 [4]. Most fractures in the elderly are caused by osteoporosis and occur from minor falls, whereas fractures in young people typically result from high-energy trauma [5]. Proximal femoral fractures among females are two to three times higher than in males [6]. Moreover, after 50 years, the possibility of sustaining a proximal femoral fracture doubles every 10 years [7]. Conservative management of intertrochanteric femoral fracture is associated with poor therapeutic outcomes, and surgical fixation is generally warranted [8]. Treatment success is influenced by various factors such as the patient's age, the type of fracture, the patient's overall health, the duration between fracture and treatment, the effectiveness of the treatment, any ongoing medical treatment, and the stability of the fixation [9]. The main purpose of the surgery is to enable the patient to start moving early, with the amount of weight-bearing being determined by the stability of the reduction [10].

Extramedullary devices with a fixed angle, like a 95-degree lag screw and side plate or blade plate, are frequently employed for internal fixation. These devices consist of a sizeable lag screw placed in the middle of the femoral neck and head, together with a side plate running alongside the lateral femur [11]. Compared to a static screw, the sliding lag screw offers the advantage of allowing for the impaction of fragments. This impaction enhances bone-on-bone contact, promoting osseous healing and reducing implant stress [12]. The problems associated with dynamic hip screw (DHS) are larger exposure, more tissue handling, and anatomical reduction, which leads to morbidity, the probability of infection and significant blood loss, and the possibility of varus collapse [13]. The bone is mechanically weakened by the side plate and screws. The instability of the fractures, osteoporosis, lack of anatomical reduction, failure of the fixation device, and incorrect placement of the lag screw in the femoral head are the common causes of fixation failure [14]. The other spectrum is the intramedullary fixation with devices like the intramedullary hip screw (IMHS), Gamma nail, antegrade trochanteric nail (ATN), trochanter fixation nail (TFN), and proximal femoral nail (PFN) [15]. The screw and side plate and blade plate have been revealed to have elevated rates of fracture union when used with fractures involving the piriformis fossa, but intramedullary nails have been suggested if the posteromedial cortical buttress cannot be established in unstable fractures [16]. The benefits of intramedullary devices include preserved blood supply to the bone fragments, less operative blood loss, and less disruption of the environment [17].

The main aim of this study is to assess the functional outcome in intertrochanteric fractures using the Harris Hip Score and the complications in intertrochanteric fractures [18-19].

## Materials and methods

This prospective observational study over two years involved operated intertrochanteric femur fracture cases. The Institutional Ethics Committee of Hinduhridaysamrat Balasaheb Thackeray Medical College and Dr. R.N. Cooper Muncipal General Hospital, where this study was conducted, issued approval (CDSCO: ECR/1654/Inst/MH/2022, DHR: EC/NEW/INST/2021/2272). Details were obtained from the clinical history proforma, and patient details were recorded in the tertiary care center.

Sample size

Sixty-six patients with intertrochanteric femur fractures were taken into the study.

Participants included in this study were all acute intertrochanteric fractures, skeletally mature patients. Those excluded were skeletally immature patients, patients with subtrochanteric extension, open fractures, old malunited fractures, trochanteric fractures associated with the neck of the femur, head of the femur, dislocation of hip and knee, and pathological fracture. All the patients were evaluated according to their general condition, and hydration and corrective measures will be undertaken accordingly. Anteroposterior and lateral radiographs of the affected hips were taken. Once the general condition was permitted, patients were taken to the operation theater. Depending on the patient's pre-operative status and the amount of blood lost during surgery, adequate blood transfusion, thromboprophylaxis, and other supportive measures were administered. Surgery (DHS/PFN/PFNA2) was decided, depending on the morphology of the fracture, affordability of the patients, facility, and experience of the operating surgeons. The incision length, surgery duration, and fluoroscopy time were recorded intraoperatively. These cases were evaluated based on the mode of injury, classification, and surgical treatment and their functional outcome with or without residual complication. The Harris Hip Score system evaluated the results of the management of intertrochanteric fractures. This system is modified according to the needs of the Indian patients. i.e., in place of “put on shoes and socks” we are using “squatting” (squatting with ease/with difficulty/unable) and in place of “sitting” we are using “cross-legged sitting” (cross-legged sitting with ease/with difficulty/unable) [20]. Roentgenographic evaluation and radiological parameters include pelvis with both hips AP view, affected hip lateral view, and shaft femur with knee joint AP/lateral views.

Data collection and statistical analysis

All the study participants were interviewed and necessary examinations were done. These findings were recorded in the case record proforma and entered in the Microsoft Excel 2013 version (Microsoft Corp., USA). The results were prepared using appropriate tables and graphs when necessary. Variations were calculated as a percentage of the total and reported. Data were reported as a percentage or mean ± standard deviation. The results were reported as proportion, and p-value < 0.05 was considered statistically significant. Data analysis was done with the help of IBM SPSS Statistics for Windows, Version 20.0 (released 2011, IBM Corp., Armonk, NY). Quantitative data were presented with the help of the mean/standard deviation/median/interquartile range (IQR). Qualitative data were presented with frequency and percentage tables, and the association among the study parameters was assessed with the help of the chi-square test with continuity correction for all 2 x 2 tables and Fisher's exact test for all 2 x 2 tables. A p-value less than 0.05 is taken as a significant level.

## Results

In the study, out of a total of 66 study patients, nine (13.64%) were treated with DHS, 38(57.58%) were treated with PFN, and 19 (28.79%) were treated with PFNA2 (Table 1).

**Table 1:** Treatment modalities used in the study DHS: dynamic hip screw, PFN: proximal femoral nail, PFNA2: proximal femoral nail antirotation 2

Treatment modality	Number of patients	Percentage
DHS	9	13.64
PFN	38	57.58
PFNA2	19	28.79
Total	66	100.00

The mean time for the duration of surgeries was the maximum for DHS (98.11 ± 13.49 minutes), followed by PFN (74.76 ± 13.94 minutes) and lastly PFNA2 (58.16 ± 6.06 minutes). Thus, the time required for the surgeries involving PFNA2 was the least, in fact, less than an hour. This association of time required for surgery completion was statistically extremely significant (Table 2).

**Table 2:** Comparison of the mean time duration for the surgeries DHS: dynamic hip screw, PFN: proximal femoral nail, PFNA2: proximal femoral nail antirotation 2

Treatment modality	Number of patients	Number of patients Meantime duration for the surgeries (minutes)	P value
DHS	9	98.11 ± 13.49	<<0.0001
PFN	38	74.76 ± 13.94	
PFNA2	19	58.16 ± 6.06	

Complications occurred in eight (9.09%) out of a total of 66 patients. The proportion of complications after surgery was the maximum in DHS with three (33.33%) patients and the least in PFNA2 patients with one (5.26%). This association of complications in the study patients with the treatment modality used was statistically insignificant (Table 3).

**Table 3:** Complications in the study patients DHS: dynamic hip screw, PFN: proximal femoral nail, PFNA2: proximal femoral nail antirotation 2

Treatment modality	Number of patients	Number of patients with post-op infections	Percentage	P-value
DHS	9	3	33.33	0.31975
PFN	38	4	10.53	
PFNA2	19	1	5.26	

In the HHE score, the improvement seen from three months to six months was statistically significant, i.e., the proportion of poor/ fair scores was decreased in each group and good/excellent scores were increased in each group. The proportion of excellent HHE was highest in PFNA2 at six months follow-up examination, which was 11 (57.19%) more than any method (Table 4).

**Table 4:** Evaluation of HHE in the different modalities PFNA2: proximal femoral nail antirotation 2, HHE: Harris Hip Evaluation

DHS HHE score result	Three months	Six months	P-value
Poor	1 (11.11%)	0 (0%)	0.035
Fair	6 (66.67%)	1 (11.11%)	
Good	2 (22.22%)	6 (66.67%)	
Excellent	0 (0%)	2 (22.22%)	
PFN HHE score result	Three months	Six months	P-value
Poor	5 (13.16%)	0 (0%)	<0.0001
Fair	27 (71.05%)	3 (7.89%)	
Good	6 (15.79%)	24 (63.16%)	
Excellent	0 (0%)	11 (28.95%)	
PFNA2 HHE score result	Three months	Six months	P-value
Poor	1 (5.26%)	0 (0%)	0.000005
Fair	14 (73.68%)	0 (0%)	
Good	4 (21.05%)	8 (42.11%)	
Excellent	0 (0%)	11 (57.19%)	

In the incidence of varus deformity, the proportion was maximum in the case of DHS with three patients (33.33%), followed by PFN with 11 patients (28.95%), and the least for PFNA2 with three patients (15.79%). Although the proportion was maximum for DHS, this association was statistically not significant. Out of 17 patients with varus deformity, six patients developed shortening, three each from PFN and DHS and none from PFNA2. These six had abdominal lurch and one patient of of DHS needed a walker for walking following six months of surgery. Of the three varus deformities in PFNA2, none of them developed any shortening of the limb or lurch (Table 5).

**Table 5:** Incidence of varus deformity DHS: dynamic hip screw, PFN: proximal femoral nail, PFNA2: proximal femoral nail antirotation 2

Treatment modality	Number of patients	Number of patients with varus deformity	Percentage	P-value
DHS	9	3	33.33	0.4820
PFN	38	11	28.95	
PFNA2	19	3	15.79	

## Discussion

In the study, out of the total 66 study patients, nine (13.64%) were treated with DHS, 38 (57.58%) were treated with PFN, and 19 (28.79%) were treated with PFNA2. The mean time for the duration of surgeries was the maximum for DHS (98.11 ± 13.49 minutes), followed by PFN (74.76±13.94 minutes) and lastly PFNA2 (58.16 ± 6.06 minutes). Thus, the time required for the surgery involving PFNA2 was the least, in fact, less than an hour. This association of time required for surgery completion was statistically extremely significant. The difference in the operative time in all three treatment groups was found to be highly significant and was probably attributed to the smaller incisions in the PFNA group. The mean duration of surgery in the study by Jangir M et al. in the DHS group was 88.25 minutes and that of the PFNA group was 68.4 minutes. Baumgaertner et al. [21] also found that the surgical times were 23% less in the PFN group in their series. In the study by Goel K et al. [22], the average intraoperative time in the DHS group was 106.40 ± 9.63 minutes, and that in the PFN group was 116.4 ± 32.67 minutes. Although this difference was not statistically significant (p = 0.149), it could be due to PFN being a relatively new method with less experience in the technique especially in the earlier cases. Pajarinen et al. [23] showed more intraoperative duration in PFN than DHS, while Saudan et al. [24] and Kumar et al. [25] showed a lesser duration in PFN as compared to DHS. In the study by Gururagavendra P [26], the average surgical time in the PFN A2 group is about 59 minutes, and in the PFN group, it is about 71.9 minutes, which was statistically significant.

Complications occurred in six (9.09%) out of a total of 66 patients. The proportion of complications after surgery was the maximum in DHS with two (22.22%) patients at least in PFNA2 patients with one (5.26%). This association of complications in the study patients with the treatment modality used was statistically insignificant. In the study by Gururagavendra P [26], complications were present in two patients (13.3%) with PFNA2 and in three (18.7%) patients with PFN.

In the HHE score, the improvement seen from three months to six months was statistically significant, i.e., the proportion of poor/ fair scores decreased in each group and good/excellent scores increased in each group. The proportion of excellent HHE was highest in PFNA2 at the six-month follow-up examination, which was 11 (57.19%) more than any method. Moreover, in our study, the mean scores for DHS were 76.22 ± 4.66 and 87.22 ± 4.29 at three and six months, respectively; the mean scores for PFN were 75.66 ± 3.92 and 87.08 ± 4.11 at three and six months, respectively; and the mean scores for PFNA2 were 76.95 ± 4.78 and 89.32 ± 3.06 at three and six months, respectively. In the study by Goel K. et al. [22], HHS for DHS at three months was 29.52 ± 5.03 and at six months was 68.48 ± 7.42, while that for PFN was 48.44 ± 6.33 at three months and 76.92 ± 6.89 at six months. Moreover, in the study by Gururagavendra P [26], the mean values of PFN and PFNA2 were 81.9 ± 7.2 and 83.6 ± 6.8, respectively.

In the incidence of varus deformity, the proportion was maximum in the case of DHS with three patients (33.33%), followed by PFN with 11 patients (28.95%) and lastly PFNA2 with three patients (15.79%). Although the proportion was maximum for DHS, this association was statistically not significant. Out of 17 patients with varus deformity, six patients developed shortening, three each from PFN and DHS and none from PFNA2. These six had lurch at six months and one patient of DHS needed a walker for walking following six months of surgery. Of the three varus deformities in PFNA2, none of them developed any shortening of the limb or lurch. 

One limitation of this study was involving numerous surgeons who had to overcome the learning curve associated with the relatively new implant.

## Conclusions

The PFN has gained wide popularity in recent years as it has been shown to withstand higher static and cyclical loading being biomechanically stronger than DHS. It acts as a buttress in preventing the medialization of the shaft. In experienced hands, it has shown less surgical exposure, less operative time, less blood loss, and early return to premorbid conditions. However, it also has its limitations with the high learning curve, high implant cost, and complications like the Z effect and reverse Z effect. The results suggest that the mean operative time was lesser in the PFNA2 group and higher values for the HHE score compared to PFN and DHS, This study concludes that these implants have a comparable radiological and functional outcome for unstable intertrochantric fracture except for less surgical time and blood loss in PFNA2. Complications such as proximal migration spiral blade into the hip joint and lateral thigh pain were not seen in this study. The superiority of one implant over another could be decisive with a still larger sample size, involving multi-center studies. In PFNA2, there is no need for another derotation screw. Innovative helical blade design provides better hold on both compact and cancellous bone, increases contact area between and femoral head, improves stability, and has lesser surgical infections and lower cases of varus deformity, thereby promoting limb shortening and zero incidences of screw cutout in the case of unstable intertrochanteric fractures. Our study showed that PFNA2 is a better implant than PFN in treating unstable intertrochantric fractures.
